# Evaluation of a comprehensive maternal newborn health intervention in rural Tanzania: single-arm pre-post coverage survey results

**DOI:** 10.1080/16549716.2022.2137281

**Published:** 2022-11-11

**Authors:** Dismas Matovelo, Maendeleo Boniphace, Nalini Singhal, Alberto Nettel-Aguirre, Jerome Kabakyenga, Eleanor Turyakira, Hannah Faye G. Mercader, Sundus Khan, Girles Shaban, Teddy Kyomuhangi, Amy J. Hobbs, Kimberly Manalili, Leonard Subi, Jennifer Hatfield, Sospatro Ngallaba, Jennifer L. Brenner

**Affiliations:** a Department of Obstetrics & Gynecology, Catholic University of Health & Allied Sciences, Mwanza, Tanzania; b Department of Pediatrics, Cumming School of Medicine, University of Calgary, Calgary, AB, Canada; c Department of Community Health Sciences, Cumming School of Medicine, University of Calgary, Calgary, AB, Canada; d Centre for Health and Social Analytics, NIASRA, University of Wollongong, Wollongong, Australia; e Institute of Maternal Newborn and Child Health, Mbarara University of Science and Technology, Mbarara, Uganda; f Department of Community Health, Mbarara University of Science and Technology, Mbarara, Uganda; g Indigenous, Local & Global Health Office, Cumming School of Medicine, University of Calgary, Calgary, AB, Canada; h Department of Public Health, Catholic University of Health and Allied Sciences, Mwanza, Tanzania; i Institute for International Programs, Department of International Health, Johns Hopkins Bloomberg School of Public Health, Maryland, United States; j Department of Preventive Services, Tanzania Ministry of Health, Community Development, Gender, Elderly and Children, Dodoma, Tanzania; k Department of Community Health, Catholic University of Health & Allied Sciences, Mwanza, Tanzania

**Keywords:** Pregnancy, Africa, delivery, obstetric, maternal health, infant health

## Abstract

**Background:**

In Tanzania, maternal and newborn deaths can be prevented via quality facility-based antenatal care (ANC), delivery, and postnatal care (PNC). Scalable, integrated, and comprehensive interventions addressing demand and service-side care-seeking barriers are needed.

**Objective:**

Assess coverage survey indicators before and after a comprehensive maternal newborn health (MNH) intervention in Misungwi District, Tanzania.

**Methods:**

A prospective, single-arm, pre- (2016) and post-(2019) coverage survey (ClinicalTrials.gov #NCT02506413) was used to assess key maternal and newborn health (MNH) outcomes. The Mama na Mtoto intervention included district activities (planning, leadership training, supportive supervision), health facility activities (training, equipment, infrastructure upgrades), and plus community health worker mobilization. Implementation change strategies, a process model, and a motivational framework incorporated best practices from a similar Ugandan intervention. Cluster sampling randomized hamlets then used ‘wedge sampling’ protocol as an alternative to full household enumeration. Key outcomes included: four or more ANC visits (ANC4+); skilled birth attendant (SBA); PNC for mother within 48 hours (PNC-woman); health facility delivery (HFD); and PNC for newborn within 48 hours (PNC-baby). Trained interviewers administered the ‘Real Accountability: Data Analysis for Results Coverage Survey to women 15–49 years old. Descriptive statistics incorporated design effect; the Lives Saved Tool estimated deaths averted based on ANC4+/HFD.

**Results:**

Between baseline (n = 2,431) and endline (n = 2,070), surveys revealed significant absolute percentage increases for ANC4+ (+11.6, 95% CI [5.4, 17.7], p < 0.001), SBA (+16.6, 95% CI [11.1, 22.0], p < 0.001), PNC-woman (+9.2, 95% CI [3.2, 15.2], p = 0.002), and HFD (+17.2%, 95% CI [11.3, 23.1], p < 0.001). A PNC-baby increase (+6.1%, 95% CI [−0.5, 12.8], p = 0.07) was not statistically significant. An estimated 121 neonatal and 20 maternal lives were saved between 2016 and 2019.

**Conclusions:**

Full-district scale-up of a comprehensive MNH package embedded government health system was successfully implemented over a short time and associated with significant maternal care-seeking improvements and potential for lives saved.

## Introduction

Globally, over a quarter of a million women die each year due to pregnancy and childbirth-related complications [1] while 2.4 million babies die within the first month of life [2]. Two-thirds (66%) of maternal deaths [1] and 38% of neonatal deaths [3] occur in sub-Saharan Africa. Tanzania has amongst the highest maternal mortality ratios in the world and persisting high neonatal mortality [1]. Most deaths occur in the intrapartum and immediate postpartum period, and could be reduced through interventions provided during routine antenatal care (ANC) [4], health facility delivery (HFD) [5], and postnatal care (PNC) [6].

Tanzania struggles with low country-wide rates of facility-based care-seeking. According to the most recent Tanzania Demographic Health Survey, only one-quarter (25%) of pregnant women attended ANC during their first trimester, half (51%) attended the recommended number of four ANC visits, over one-third (36%) of women reported delivering outside a health facility, and only one-third (34%) reported PNC within 48 hours following delivery [7].

Low maternal care-seeking in Tanzania is influenced by factors on both the ‘service’ provision side and on the ‘demand’ side. Common facility-based basic emergency obstetric and newborn-care (BEmONC) service-side barriers include lacking clinical guidelines, essential medicines, equipment, and staff training opportunities [8]. Service provision gaps are not evenly distributed; rural facilities score significantly lower on BEmONC ‘readiness’ compared to urban counterparts [8]. This is especially true in northwestern regions surrounding Lake Victoria where facilities struggle with inadequate infrastructure and supplies plus significant transportation and training gaps [9,10]. Perceived low quality of women-health worker interactions and disrespect towards pregnant women aggravates uptake of routine ANC and facility-based delivery services [11,12].

On the demand side, sociocultural factors involving families and communities are often at the root of women’s choice to seek facility-based care and are frequently underpinned by social determinants of health. In Tanzania, cited care-seeking barriers include low-socioeconomic status [11–14], lacking household support especially from male household heads [15,16], familial power imbalances [17], long distances to facilities [14,18], and lacking availability of means for travel [14]. Such barriers are exacerbated for adolescents [17] and women with low literacy [19].

Reducing maternal and newborn mortality in Tanzania requires scalable, system-wide, and community-oriented interventions that address these complex service- and demand-side barriers [16,20,21], while leveraging existing policy. In response, the *Mama na Mtoto* (Swahili for ‘mother and child’) initiative (2016–2020) adapted a comprehensive intervention package developed in Uganda [22,23] for use in Lake Zone, Tanzania. Through a South-South-North coalition (Catholic University of Allied and Health Sciences (CUHAS) and Council Health Management Teams (Tanzania); Mbarara University of Science and Technology (Uganda); University of Calgary, Agriteam Canada, Save the Mothers, and Canadian Paediatric Society (Canada)), partners worked to increase district-wide capacity and demand for ANC, delivery services, and PNC.

*Mama na Mtoto* was funded by Global Affairs Canada through two grants – an implementation grant and a separate International Development and Research Centre-administered grant which supported evaluation. *Mama na Mtoto* evaluation had two objectives: (1) Assess *Mama na Mtoto* package ‘effectiveness’ [24] in improving maternal, newborn health (MNH) outcomes using mixed methods; and (2) Assess and document its implementation (process evaluation) using the *RE-AIM* (Reach, Effectiveness, Accessibility, Implementation, Maintenance) framework [25].

This paper presents key quantitative ‘effectiveness’ results from a rigorous MNH coverage survey conducted before and after implementation of the *Mama na Mtoto* intervention package in Misungwi District, Tanzania.

## Methods

### Study design

**Figure 1.**
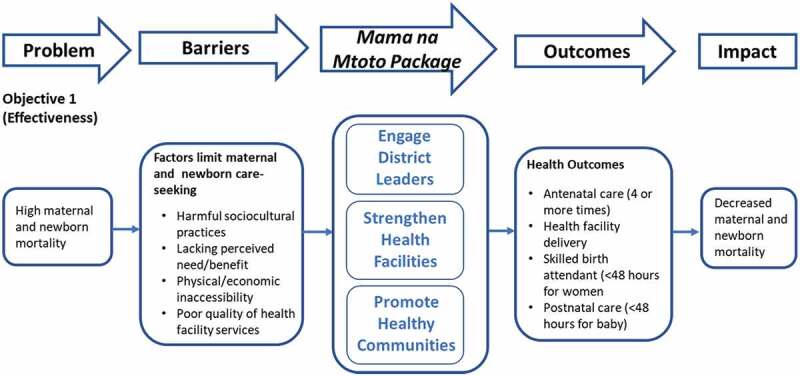
Mama na Mtoto logic model showing the two research objective pathways.

This quantitative study was embedded within a larger mixed-methods implementation study evaluating the *Mama na Mtoto* intervention using an effectiveness-implementation hybrid design (Type II; [24]). Figure 1 illustrates our study objectives related to effectiveness showing key outcome measures. This prospective single-arm pre-post intervention trial survey (Objective 1) adopted methodology and tools from the ‘*Real Accountability: Data Analysis for Results (RADAR)* toolkit, created to support evaluation of real-life MNH implementation projects [26]. Our baseline survey served as a *RADAR* Coverage Survey pilot, providing feedback to now-available online resources [27,28].

### Study area

*Mama na Mtoto* was implemented in Misungwi and Kwimba Districts, Mwanza Region, Tanzania. District selection was purposive, influenced by low-achieving MNH indicators, longstanding relationships with Bugando Medical Centre (a zonal referral and teaching hospital), expressed district leadership interest, and Mwanza Regional Health Management Team input. Implementation involved two districts during the three and a half-year project period; however, this pre-post coverage survey evaluation component only occurred in Misungwi, facilitating larger population proportion sampling while accommodating timing, budget, and logistical constraints.

Misungwi, a predominantly rural district, had a population of 396,055 (2016) [29] with subsistence farming, fishing, and small-scale animal rearing as main livelihood sources. Health facilities comprised two hospitals, four health centres, and 39 dispensaries, largely government-owned (91%).

### Intervention

*Mama na Mtoto* in Tanzania involved a comprehensive package of activities providing general health system strengthening whilst encouraging specific MNH capacity and motivation. The intervention was informed by experiences from *MamaToto*, a 2012–2015 maternal child health intervention in southwest Uganda [23]. Through extensive consultation and ongoing planning engagement with district and regional health leaders, activities were adapted based on Government of Tanzania priorities, policies, and guidelines, accommodating for cultural and district health system context. Implementation was structured according to *MamaToto*-informed best practices articulated within the ‘*Maximizing Engagement for Readiness and Impact (MERI) Approach’* [30], including implementation strategies, a process model (to guide steps of implementation), and a motivational framework, intended to stimulate positive perceptions around the intervention and foster district, facility and community leadership.

**Figure 2.**
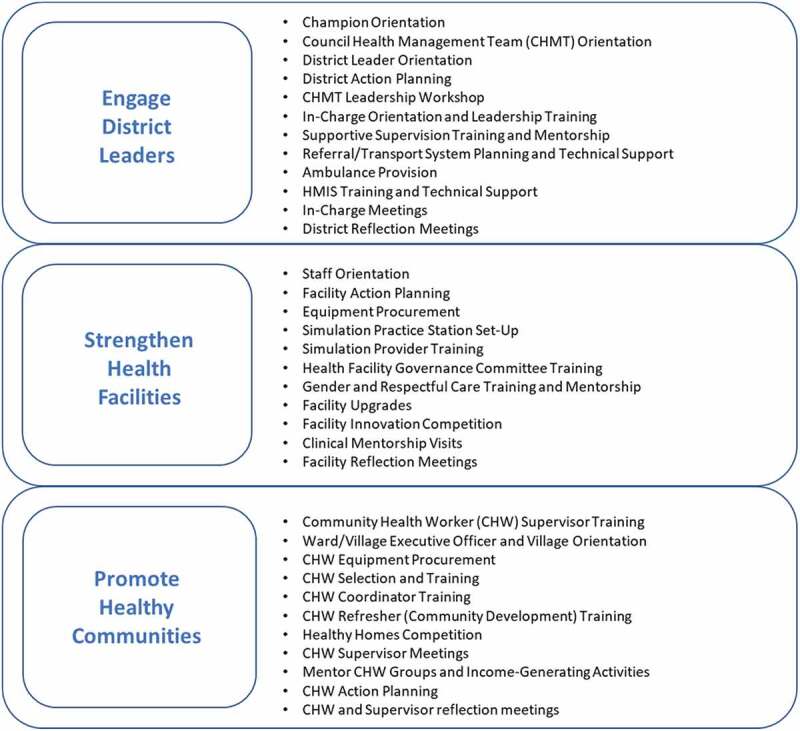
Mama na Mtoto package activities.

Activities occurred at three levels: (1) district; (2) health facility; and (3) community. Figure 2 details the broad intervention bundle applied at each level including meetings, training, equipment provision, facility upgrades, technical assistance, and mentorship delivered in Misungwi over 30 months. The Misungwi Council Health Management Team set their own MNH priorities. Activities followed government policies, guidelines, and curriculum while using existing health system structures. District health managers participated in leadership training and led facility-based quality improvement, supportive supervision, and planning initiatives. Hands-on, simulation-based workshops and practice sessions refreshed clinician BEmONC skills. Data management training and mentorship promoted Health Management Information Systems (HMIS) capacity. Workshops for existing Health Facility Governance Committees (HFGC) emphasized committee member roles and led to facility MNH action planning. Training and supervision encouraged a strong network of volunteer Community Health Workers (CHWs) to conduct home visits, assess and triage pregnant women and newborns, and mobilize their communities for health innovations and income-generating activities. In total, 150 health providers, 775 CHWs, and 927 managers and HFGC members were trained. All health facilities received key MNH equipment; infrastructure upgrades occurred at three sites. The delivery of activities followed a process model that involved seven purposeful steps: Scan, Orient, Plan, Equip, Train, Act, and Reflect (*SOPETAR*) [30]. To stimulate uptake and leadership, implementation was designed to align with government programming, integrate within existing health system structures and cultivate self-reliance, transparency, and collective action.

### Study indicators

Primary outcomes assessed were: Pregnant women attending ANC at least four times (ANC4+); skilled birth provider at delivery (SBA); receiving PNC within 48 hours after delivery (PNC-woman) (Supplementary Material A). Reported deliveries at a health facility (HFD) and newborns receiving PNC within 48 hours after delivery (PNC-baby) were secondary indicators. Participant characteristics collected included age, hamlet residential type (urban, mixed, rural), marital status, education level, ethnicity, religion, and parity. These indicators are defined as per the RADAR tool methodology [27].

### Sample size determination

Given the summary statistic of the indicators are proportions ‘p’, sample size calculation used the formula n = deff*(1.96 + 0.84)^2^*(p_*2*_*q_2_ + p*_*1*_*q*_*1*_*)/d*^*2*^ [31] for each primary outcome indicator. In this formula, ‘*p*_*1*_’ and ‘*p*_*2*_’ are the indicator proportions estimated at times 1, 2, respectively, and *deff* is the design effect that considers the clustered nature of the data. Baseline indicator percentage and fertility rate estimates used Mwanza Region Tanzania Demographic Health Survey results [7]. Indicator targets were determined with district and coalition partner input. Design effect estimates were based on observed values for the same indicators from a previous coverage survey during the *MamaToto* intervention in Uganda. Targets aimed for 80% power and a significance level alpha of 0.05. Based on calculated outputs (Supplementary Material B), we aimed to recruit 2,000 households to ensure sufficient eligible women participants from surveyed households.

### Sampling procedure

A two-stage stratified cluster sampling method was used to identify unique households. At the first stage, hamlets (smallest identifiable geopolitical units) served as clusters, stratified by residential type (rural/mixed/urban) according to reported district proportions [29]. The =RAND() function in MS Excel was used for randomization; a total of 67 out of 724 hamlets were selected in the district: two urban, 25 mixed, and 40 rural.

Second-stage sampling of individual households within hamlet clusters used a ‘*wedge sampling’* protocol (Supplementary Material C) designed as an alternative to full enumeration of household clusters, involving a non-probability (purposeful) sample. In target communities at baseline, accurate census household registration and high-resolution Global Positioning System (GPS) maps were not available for most communities. Misungwi district had difficult topology, remote and scattered households, and heavy seasonal rains, which made enumeration for a complete sampling frame infeasible within time and budget constraints.

The ‘*wedge sampling*’ protocol involved trained ‘mappers’ starting at the estimated geographic centre of the hamlet and walking towards the hamlet boundary/perimeter based on a randomly selected ‘directional’ line (N, NE, E, SE, S, SW, W, or NW). Accompanied by community leaders, mappers walked back to the centre and then back to the perimeter in a continued zigzag pattern to cover a ‘wedge’-like area of the hamlet, mapping an area like the space between two bicycle spokes. All households in proximity within this wedge were approached until 30 eligible households (consistent with *RADAR* recommendations and realistic number of households that could be surveyed in a day) were successfully enumerated and introduced to the study. This *wedge sampling* process encouraged the possibility of inner cluster and outer cluster households to be sampled, hence avoiding sampling only households close to one cluster point. A ‘household’ was defined as a group of persons residing in the same housing dwelling with common cooking/eating arrangements, recognizing one member as the household head. Hamlets with fewer than 45 total households were enumerated entirely. Eligible enumerated households were later approached by an interviewing team for data collection. See Supplementary Material C for more protocol details.

The same hamlets (‘clusters’) and *wedge sampling* protocol were used at baseline and endline, but with newly random-generated directional lines. Therefore, this process, in addition to potential household relocation and new households appearing within hamlets, did not guarantee that the exact same households were visited pre and post.

### Data collection

Data were collected using a structured questionnaire during September–October 2016 (baseline) and September–October 2019 (endline). The *RADAR Coverage Survey* aligns with key global MNH indicators and demographic characterization, with features similar to the *Demographic and Health Survey* and *Multiple Indicator Cluster Survey* [27]. *RADAR* tools used were the *Household Questionnaire*, *Woman’s Questionnaire*, and *Under-Five Questionnaire;* however, indicators for this paper used data from only the Woman’s Questionnaire.

Surveys were translated from English to Swahili and converted into electronic format using *Open Data Kit* (ODK) [32]. Tools were field pilot-tested with minor adjustments to field protocol, questions, and response options accommodating local context.

Trained data collectors verbally administered survey questionnaires to eligible household members using *ODK Collect* application on tablet devices (Asus ZenPad-7, Samsung Galaxy Tab A-7). Eligible women aged 15–49 years old, and caretakers of children aged 0–5 years old were interviewed if they were usual residents of the household or slept in the household the night before. Women or caretakers with severe illness, disabilities, or issues with competency that impaired them from completing the survey were excluded. Non-Swahili speaking participants were assigned a Sukuma (study area dialect)-speaking interviewer; translation to Sukuma was done verbally at the time of interview.

Where eligible participants were not successfully interviewed on a first attempt, at least two ‘call-back’ attempts were made. At baseline, two call-backs occurred later the same day, then a third attempt was made several weeks later. Given low response rate of baseline survey completion during the third attempts, endline call-backs were limited to two same/next-day attempts to conduct the interview only.

### Data management and analysis

Regular review of completed questionnaires plus intermittent spot-checks and re-interviews by field supervisors ensured data quality and protocol compliance. Data were downloaded daily onto an *ODK* server then exported to statistical analysis software. Data merging, cleaning, and analysis used *R* version 3.3.2 [33] and *STATA* version 15 [34] according to pre-determined indicator definitions.

Frequency for each outcome indicator were calculated according to indicator definitions (Supplementary Material A). Weighted point estimates and confidence intervals were calculated for each indicator. Sampling weights for each cluster were assigned by calculating the inverse probability of selection of a household within each cluster in the stratum. Numbers of total households per cluster for weighting data were estimated by local leaders during mapping. Weights were adjusted for non-response then standardized by dividing each weight by the mean of all the weights. Data analysis used *Taylor linearization* [35] to adjust standard errors for cluster effects. Design effect used *deff = 1+ (m-1) ICC*, where *m* is the average cluster size sampled and intra-cluster correlation coefficient (*ICC)* value is calculated for each indicator.

The *Lives Saved Tool* (*LiST*) was used to predict intervention impact. *LiST* is based on a deterministic, linear, mathematical model; the *LiST sub-national Wizard* incorporates intervention coverage rate changes and available country/region datasets to estimate deaths averted and mortality rate changes [36]. Entered data used projected 2016 Misungwi population according to the most recent (2012) national census [29], total fertility rate as per Tanzania Demographic Health Survey Mwanza region data, along with study baseline (2016) and endline (2019) ANC4+ and HFD frequencies. The ‘interpolate’ function automatically generated 2017 and 2018 estimates. Notably, *LiST* did not provide options to produce estimates based on SBA, PNC-woman, or PNC-baby frequencies.

### Ethics and study approval

This study was approved by the CUHAS/Bugando Medical Centre Ethics and Review Committee (CREC/070/2015), University of Calgary Conjoint Health Research Ethics Board (REB15–1919), and National Institute for Medical Research Lake Zone Institutional Review Board (MR/53/100/400) and registered at ClinicalTrials.gov (#NCT02506413). All participants provided signed informed consent; thumbprint was accepted for those unable to write.

## Results

### Sociodemographic characteristics

A total of 1,977 households at baseline and 1,835 households at endline participated in the survey. The majority of households were from rural areas (baseline: 55.8%, endline: 56.8%). Within these households, 2,438 women completed the *Women’s Questionnaire* at baseline with 2,073 women respondents at endline. Survey response rates were 92.5% (baseline) and 86.0% (endline); at baseline, the majority of non-responders were on an ‘extended absence’ (53.4%) and at endline were ‘not at home’ (63.6%).

Table 1 shows main demographic characteristics of women respondents for each data collection period. Mean participant age was 28.3 (SD: 9.5) years old at baseline and 29.0 (SD: 9.9) years old at endline. Participants were largely of Sukuma ethnicity (baseline: 93.5%, endline: 91.9%); the majority reported Christian religion (baseline: 85.1%, endline: 83.8%). Most women were primary school educated (baseline: 68.2%, endline: 62.8%). A large majority reported having ever given birth (baseline: 80.8%, endline: 78.5%); mean number of births per respondent were 3.5 pre- and 4.6 post-intervention.

**Table 1.** Socio-demographic characteristics of participants at baseline and endline.

Characteristic	Baseline n = 2,431 (weighted)	Endline n = 2,070 (weighted)
	Frequency (%)	Frequency (%)
Age (in years)		
15-19	505 (20.8%)	451 (21.8%)
20-24	509 (20.9%)	374 (18.1%)
25-29	374 (15.4%)	331 (16.0%)
30-34	355 (14.6%)	313 (15.1%)
35-39	257 (10.6%)	194 (9.4%)
40-44	274 (11.3%)	216 (10.5%)
45-49	158 (6.5%)	191 (9.2%)
Mean Age (SD)	28.3 (9.5)	29.0 (9.9)
Location		
Rural	1,383 (56.9%)	1,175 (56.8%)
Mixed	664 (27.3%)	482 (23.3%)
Urban	384 (15.8%)	413 (19.9%)
Marital status		
Married/Living in a union	1,566 (64.4%)	1,392 (67.3%)
Not in a Union	865 (35.6%)	678 (32.7%)
Education level		
No school	458 (18.8%)	401 (19.4%)
Primary	1,657 (68.2%)	1,300 (62.8%)
Secondary	296 (12.2%)	323 (15.6%)
Higher	20 (0.8%)	46 (2.2%)

### Maternal health characteristics and care-seeking practices

Of women reporting prior births, slightly over half cited a birth within two years of survey (baseline: 52.8%, endline: 53.3%). Key indicator changes are presented in Table 2.

**Table 2.** Key maternal newborn care-seeking indicators, baseline and endline.

		Baseline n = 1,032		Endline n = 868			
Indicator		% [95% CI]	Design effect	% [95% CI]	Design effect	Absolute % Change [95% CI]	p-value
Primary	ANC 4+	47.1 [42.2, 52.0]	2.6	58.7 [54.9, 62.3]	1.3	+11.6 [5.4, 17.7]	<0.001
	SBA at delivery	63.7 [59.0, 68.2]	2.2	80.3 [76.8, 83.4]	1.5	+16.6 [11.1, 22.0]	<0.001
	PNC-woman	42.0 [37.8, 46.3]	2.0	51.1 [46.9, 55.3]	1.6	+9.2 [3.2, 15.2]	0.002
Secondary	HFD	61.1 [56.7, 65.4]	2.2	78.3 [74.1, 82.1]	2.1	+17.2% [11.3, 23.1]	<0.001
	PNC-baby	50.2 [45.4, 55.0]	2.5	56.3 [51.6, 60.9]	2.0	+6.1% [−0.5, 12.8]	0.07

CI= confidence interval.

Nearly, all these women reported attending at least one ANC visit (baseline: 92.5%, endline: 90.1%). The mean number of ANC visits at baseline was 1.46 (SD: 1.94) and at endline was 3.95 (SD: 1.35). A small proportion of women (baseline: 14.0%, endline: 20.0%) reported attending ANC within their first trimester (i.e. before 12 weeks gestation). A statistically significant absolute increase occurred when comparing endline (58.7%) and baseline (47.1%) results for ANC4+ (+11.6, 95% CI [5.4, 17.7], p < 0.001).

Most women delivered at a health facility (baseline: 61.1%, endline: 78.3%) which was a significant increase (+17.2%, 95% CI [11.3, 23.1], p < 0.001). A skilled birth attendant was reported to be present at 63.7% of deliveries at baseline which significantly increased to 80.3% at endline (+16.6, 95% CI [11.1, 22.0], p < 0.001). A small proportion of women reported delivery by Caesarean section (baseline: 8.4%, endline: 5.2%).

Postnatally, 42.0% of respondents’ pre-intervention and 51.1% post-intervention reported care for themselves within 48 hours after delivery (+9.2, 95% CI [3.2, 15.2], p = 0.002); an increase in PNC-baby (+6.1%, 95% CI [−0.5, 12.8], p = 0.07) was observed, however, was not statistically significant.

### LiST *results*

*LiST* results produced estimates according to ANC4+ and HFD coverage changes between baseline and endline. Based on entered assumptions, modelling predicted 121 neonates, 67 children aged 1–59 months, and 20 maternal lives saved in the target district between project start (2017) and end (2019). Maternal mortality rate was estimated to decrease from 112.8 to 100.0 per 100,000 with an estimated decline in neonatal mortality rate from 23.3 to 21.6 per 1,000 live births.

## Discussion

In rural Tanzania, a district-wide, comprehensive MNH package simultaneously addressing service and demand barriers was associated with significantly increased frequency of facility-based ANC, delivery, and PNC. The *Mama na Mtoto* intervention (2017–2019) replicated an implementation approach and MNH package from Uganda, adapted for Tanzania context and policy, documenting scalability and demonstrating potential MNH impact. This study provides important evidence that district-wide, integrated MNH programming can be scaled-up and produce outcomes towards maternal and newborn survival over a relatively short time.

Two published intervention studies from East Africa similarly demonstrate effectiveness of comprehensive, district-level activity ‘bundles’ in improving MNH outcomes. In Eastern Uganda, the *MANIFEST* study (2013–2015) involving CHW mobilization, facility BEmONC training, supportive supervision and management training throughout districts, resulted in positive effects on early ANC and newborn-care practices [37]. *Saving Mothers, Giving Life*, a large, five-year, multi-partner effort in Uganda and Zambia used a district-wide health system strengthening approach, employing a package of supply and demand-side maternity-care interventions, documenting higher rates of facility deliveries [38] and significant (40%) maternal mortality decline in project facilities and districts [39]. Our study adds further evidence of potential for a system-wide, comprehensive, integrated MNH package to impact health outcomes.

During Ugandan *MamaToto* experiences, package ‘comprehensiveness’ and ‘embeddedness’ were identified as key success factors. Post-*Mama na Mtoto* intervention, stakeholders credit these same attributes to Misungwi community success. We attribute emergence of these qualities largely to our implementation process, especially our process model (Figure 2), which works towards implementation strength. Systematic analyses have documented broad activities and integration within existing structures and systems as common features amongst effective MNH interventions [40–42]. Additionally, ‘embeddedness’ is a characteristic associated with scalability and sustainability beyond the donor-funded period [39,43,44]. Indeed, following this intervention, Misungwi health structures (CHWs, CHMT, HFGC) continue to be in place and prepare to meet emerging needs during the recent COVID-19 pandemic. Supportive mentorship and shared learning continue between district health facility, community teams, and CUHAS-Bugando technical team members who interact regularly, owing to their class of care-referral relationship.

Stakeholders affirmed additional *Mama na Mtoto* success factors that were core to the *MERI Approach* [30] which guided implementation. **At the district level**, active leader engagement, full-district (not piecemeal) implementation, leadership capacity development, strengthened supportive supervision, and alignment with pre-existing district processes were identified as vital for success, consistent with expert panel recommendations for ensuring health system leadership engagement [40,45]. **At health facilities**, simulation training, peer-to-peer learning, equipment/infrastructure assurance, and broad staff and HFGC engagement were identified as key activities and strategies. **In communities**, investment in a high-density, district-wide, district-owned volunteer CHW network was credited with overcoming demand-side barriers, consistent with findings from global systematic reviews which provide evidence for CHW effectiveness in providing MNH education [46], improving neonatal health [47], and improving maternal health indicators [41].

## Limitations and strengths

Given the absence of a comparison group in our study, we cannot attribute, in full or in part, coverage indicator improvements documented in this survey to the *Mama na Mtoto* intervention specifically. Other initiatives and government programming in Misungwi during 2017–2019 likely contributed to positive MNH outcome change. While Misungwi had no other major MNH-focused projects, *Results-Based Financing* [48] was a national program active for some months during the intervention period, which added positively to service delivery quality, complementing *Mama na Mtoto* activities through provision of nominal funds to motivated facilities, some of which were used to address MNH-gaps.

Our study balances scientific integrity with ‘real-life’ practicalities. We used a standardized tool (*RADAR)*, rigorous interview methodology, and our sample size was powered to detect changes. We justify use of a single-arm trial for practical and ethical reasons; given country and donor interest in programming in the study region, nearby districts were likely exposed to MNH initiatives that our intervention would not; dividing our target district for staged implementation did not fit with our ‘full district’ approach; available funding timelines (total 3.5 years) could not accommodate a two-district step-wedge design. ‘Far-away’ comparison districts were deemed too dissimilar (ethnicity/language/setting), too costly, and unethical given unlikely opportunities for future intervention. This decision was very carefully considered and significantly informed by decision-makers. To strengthen our study design, our larger effectiveness study incorporated followed qualitative components and tracked HMIS district data trends to strengthen confidence in relating results to our intervention [49].

We also acknowledge limitations related to sampling methods. While stratified random sampling is *RADAR*-recommended [50] and the current gold standard, modifications were necessary to accommodate financials, resources, timing, and logistical constraints at baseline, which often deter implementers from undertaking quality data collection [28]. At the first stage, our modified ‘wedge sampling’ approach used systematic random sampling stratified by urban/rural status; however, full hamlet listings were required to ensure proportionality to population size, which we were unable to attain. At the second stage, random GPS directions provided variability as a substitute for systematic random sampling of households from a household list, which were not available or accurate. Third-stage sampling used the gold standard approach of interviewing all eligible women in the household.

Our results had potential for underestimation of endline changes for key indicators. The *Mama na Mtoto* intervention period was short (30 months); endline coverage data collection occurred just weeks following final intervention activities. CHW sensitization, facility support, and district capacity activities did not all start on day one but rather progressively ramped up during the intervention period. Our study tool queried births occurring in the past 24 months (and hence pregnancies beginning up to nine months earlier i.e. 33 months prior to survey); this resulted in our surveying some women whose antenatal, delivery, and postpartum care would have occurred prior to potential *Mama na Mtoto* intervention exposure and hence, potential for underestimates of the actual change. Furthermore, true intervention outcome changes may take time; demand-side behaviour change may require multiple contact points and family, community and culture change can take years. In support of delaying evaluation, Zivetz et al. [51], recommend postponing post-project impact assessments by at least two years following intervention end to improve accuracy. Additionally, *LiST* impact estimates based on indicator coverage pre and post differences may have produced underestimates, since calculations were only able to incorporate two of our five key indicators. That said, *LiST* has limitations in the assumptions it makes; however, it gives a flavour, based on evidence for the scope of change and potential for real life impact, reminding us of how care seeking change and impact may be related.

Importantly, this coverage survey had purpose and provided value beyond academic publication. Baseline results helped articulate gaps, support intervention planning, engage stakeholders, and direct district-led priority setting. As Misungwi district is not part of usual Tanzania Demographic Health Survey sampling, stakeholders and decision-makers highly valued the unprecedented quality and quantity of MNH data which enabled more targeted planning and activities. At endline, broad dissemination of largely positive MNH results sparked community, facility, district and regional-owned celebrations of success (irrespective of attributed source) and prompted new planning and priority setting towards *Mama na Mtoto ‘*sustainability phase’ transition (i.e. beyond partner funding).

## Conclusion

In our setting, integrated MNH programming was successfully implemented throughout an entire district over a relatively short time. *Mama na Mtoto* exemplifies ‘spread’ scale-up (from the Ugandan to Tanzanian setting) and was associated with significant health outcome improvements and saved lives that occurred in Misungwi during the same interval. We believe *Mama na Mtoto* sustainability was enhanced through active district leadership, highly engaged communities and health facilities with a collective voice, and activity embeddedness within existing health structures.

*Mama na Mtoto* provides a model readily available for adoption by policymakers, implementing partners, and funders. We hypothesize that *Mama na Mtoto* has potential for impactful adaptation and further spread within other regions with high maternal and newborn deaths in Tanzania and elsewhere in East Africa and even globally within additional technical health programming areas. Implementers from within districts themselves, those designing projects with national investment or donor support, however, small or large the catchment or the location should consider the value of integrated and comprehensive programming for its potential impact.

Accelerating progress towards saving the lives of women and babies in Tanzania, Sub-Saharan Africa, and globally requires scalable solutions that address both service and demand gaps. An integrated district-led, district-wide, health systems approach has potential to avert deaths, maintain results over time, and enable capacity to meet emerging health needs.

## Supplementary Material

Supplemental Material

## Paper context

Lives of babies and mothers in Sub-Saharan Africa can be saved through high-quality facility-based care accessed before, during and after delivery. In rural Tanzania, a full-district, comprehensive maternal newborn health package embedded within the government system was successfully implemented over a relatively short time and associated with significant increases reported antenatal, delivery and postnatal care-seeking. This scalable model demonstrates potential for saving lives and broader adoption.
