# Supplementary material for: Evaluation of a comprehensive maternal newborn health intervention in rural Tanzania: single-arm pre-post coverage survey results
Source: Glob Health Action. 2022 Nov 11;15(1):2137281. doi: 10.1080/16549716.2022.2137281 (PMC9665093; doi:10.1080/16549716.2022.2137281)
Supplement: Supplemental Material [file ZGHA_A_2137281_SM2525.zip › Supplementary_Material_A_03_DEC_2021.docx]

*Supplementary Material A*

**Key Indicator Definitions**

***Mama na Mtoto* Coverage Survey, 2016 and 2019**

**Antenatal care four or more visits (ANC4+)**

- Definition: Percent of women attended ANC at a health facility at least four times during pregnancy by any provider for reasons related to the pregnancy.
- Numerator: Number of women 15-49 years of age with a live birth in the 2 years prior to the survey attended ANC at a health facility at least four times during for reasons related to the pregnancy
- Denominator: Total number of women aged 15-49 years surveyed with a live birth in the 2 years prior to the survey
- Notes:
  - Women who reported at least four ANC visits at a health facility.
  - For women with more than one live birth in the two years preceding the survey, this refers only to the most recent live birth.

**Skilled birth attendant at delivery (SBA)**

- Definition: Percentage of births attended by skilled health personnel
- Numerator: Number of live births to women aged 15-49 years in the 2 years prior to the survey attended during delivery by a skilled health professional.
- Denominator: Total number of women aged 15-49 years surveyed with a live birth in the 2 years prior to the survey.
- Notes:
  - Woman reported doctor, nurse, midwife, clinical officer assisted at delivery.
  - Review text in “Other” category response and classify whether the response is a “skilled attendant” based on country context.
  - For women with more than one live birth in the two years preceding the survey, this refers only to the most recent live birth.

**Postnatal care for woman (PNC-woman)**

- Definition: Percentage of mothers who received postnatal care at a health facility within two days of childbirth
- Numerator: Number of women aged 15-49 years with a live birth in the 2 years prior to the survey who received postnatal care at a health facility within 2 days of birth.
- Denominator: Total number of women aged 15-49 years with a live birth in the 2 years prior to the survey.
- Notes:
  - A. Facility delivery: Mother received health check before leaving the facility or after she left the facility.
  - B. Non-facility delivery: Mother received health check before leaving the facility.
  - The numerator includes both A and B as described above.
  - For women with more than one live birth in the two years preceding the survey, this refers only to the most recent live birth.

**Health Facility Delivery (HFD)**

- Definition: Percentage of births delivered at a health facility
- Numerator: Number of women 15-49 years of age with a live birth in the 2 years prior to the survey who delivered at a health facility
- Denominator: Total number of women aged 15-49 years surveyed with a live birth in the 2 years prior to the survey
- Note:
  - Denominator consists of the most recent live birth that occurred in the last 2 years. This is to ensure that the women only provides information about deliveries that occurred during the project.

**Postnatal care for baby (PNC-baby)**

- Definition: Percentage of babies who received postnatal care at a health facility within two days of birth
- Numerator: Number of women 15-49 years of age with a live birth in the previous two years where the newborn received a postnatal care visit at a health facility within two days of birth
- Denominator: Number of women 15-49 years of age with a live birth in the previous two years.
- Notes:
- A. Facility delivery: Newborn received health check before leaving the facility or after newborn left the facility.
- B. Non-facility delivery: Newborn received health check before leaving the facility.
- The numerator includes both A and B as described above.
- For women with more than one live birth in the two years preceding the survey, this refers only to the most recent live birth.
