# Supplementary material for: Evaluation of a comprehensive maternal newborn health intervention in rural Tanzania: single-arm pre-post coverage survey results
Source: Glob Health Action. 2022 Nov 11;15(1):2137281. doi: 10.1080/16549716.2022.2137281 (PMC9665093; doi:10.1080/16549716.2022.2137281)
Supplement: Supplemental Material [file ZGHA_A_2137281_SM2525.zip › Supplementary_Material_B_03_DEC_2021.docx]

*Supplementary Material B*

**Sample size estimation, *Mama na Mtoto* Coverage Survey**

| Indicator* | Estimated Baseline % | Estimated Endline % | Difference | Design effect | Required number of children/women per survey | Required number of households per survey |
| --- | --- | --- | --- | --- | --- | --- |
| ANC4+ | 42% | 60% | 15% | 2 | 337 | 1,338 |
| SBA | 54% | 65% | 12% | 1.7 | 440 | 1,749 |
| PNC-woman | 20% | 35% | 11% | 1.5 | 377 | 1,495 |

*Denominator (target children): youngest child, last birth in last 2 years.
