# Supplementary material for: Evaluation of a comprehensive maternal newborn health intervention in rural Tanzania: single-arm pre-post coverage survey results
Source: Glob Health Action. 2022 Nov 11;15(1):2137281. doi: 10.1080/16549716.2022.2137281 (PMC9665093; doi:10.1080/16549716.2022.2137281)
Supplement: Supplemental Material [file ZGHA_A_2137281_SM2525.zip › Supplementary_Material_C_03_DEC_2021.docx]

*Supplementary Material C*

***Wedge Sampling’* Enumeration and Mapping Protocol**

***Mama na Mtoto* Coverage Survey, September 2016**

**Background and approach:**

General Approach: Sampling will be conducted using a 2-stage cluster design to identify a total of 2000 unique households (as per sample size calculation) for the baseline survey. The initial sample identified will be stratified by hamlet type (rural/urban/mixed) according to proportion in district. Sixty-seven hamlets for sampling will be selected by a random list function. Within these, a wedge sample will be ‘swept’ which is the lowest cluster unit. Each wedge will comprise at least 30 households and will serve as a non-probability (purposeful) sample. Note that weighting of surveys may occur at the analysis stage to account for differences in total estimated households/women in the hamlet and design effect (DE) will be calculated for each indicator.

Each wedge will comprise the 30 households with closest proximity to a directional line using perpendicular access using an area like a bicycle ‘spoke,’ encouraging most equal probability of sampling by all households. The directional line will be the one which transects from the approximate geographic centre (middle) of the hamlet through to its perimeter/boundary according to a randomly selected direction. The direction will be randomly determined for each hamlet using a random number selection, using numbers 1-8, with directions assigned according to an equal distribution categorized into 8 possible starting line directions (N, NE, E, SE, S, SW, W, NW).

This strategy involves advance mapping which will be accompanied by sensitization of the selected households. GPS documentation will be a key part of the process and specific steps of sensitization will be documented. As a check and to document the process, GPS coordinates of the directional line and households will be documented. As well, notes re logistics and timing will be noted to ensure accurate repeat samples at endline and to better understand potential future use/adaptation of this process.

**When might this sampling/sensitization process be most helpful?**

Wedge sampling provides an alternative to Stratified Random Sampling (SRS; current gold standard for cluster surveys) where household lists/census data is not available and difficult to obtain, there is a limited budget for detailed mapping, or in large geopolitical clusters especially with remote populations or difficult topology, poor access to GPS maps/photos without boundaries. This approach may also be helpful to use in potentially politically unstable areas.

Assumptions: Household (HH) lists are not available/accurate; there is some degree of heterogeneity even within clusters; reliable, trained Research Assistants (RAs) are used who understand mapping and are well-supervised; local leaders (i.e. Village Executive Office-VEO, and hamlet leaders) support the study and are willing to cooperate in enumeration plus have capacity to accurately identify geopolitical boundaries and an approximate centre (i.e. hamlet middle) for the second stage area. We also assume that ‘bands’ moving outward from the geographic centre (i.e. in selected hamlets) are somewhat homogenous in terms of dispersement of the population (such as extending from the ‘bullseye’ of a target outward).

Potential bias/limitations vs SRS: Non-probability design; potential bias of central vs. peripheral clustering (depending on variable being measured, such as prevalence of a specific disease like diarrhea which may be more likely to be clustered centrally); errors in proper sampling (hard to ensure objectivity) even according to wedge guidelines such as for centre of sample area (hamlet centre), skipping households, walking in correct direction, GPS/usage errors); potentially less able to assess prevalence in an entire population. Weighting in analysis is critical as sampling is not done proportionally with respect to the size of the hamlet. An accurate estimate of the total number of households is needed in weighing for analysis. Theoretically, if population is fast-growing, this may reduce accuracy (though not proven).

Main Advantages vs SRS: Cost and time reductions for mapping; well documented use for monitoring trends in prevalence over time and pre/post as long as same clusters used—can reduce some bias through careful supervision and some other steps listed.

How does this compare to Expanded Programme of Immunization (EPI) sampling? Overall, this process has some features of EPI-sampling though incorporates additional steps in an effort to reduce bias. It involves a larger sample size than EPI (EPI uses 30 x 7); uses advanced mapping with trained mappers; involves close supervision, GPS checks; spreads clusters through an entire wedge (i.e. along the full length of a line), not just closest to an initial HH; EPI starts at most populated centre, this uses the geographic centre as a starting point; employs call backs which EPI does not; EPI looks at all children in a household- most of our key indicators focus on youngest children only, therefore less clustering within a single home.

**Mama na Mtoto Wedge Sampling, mapping and enumeration details and logistics:**

Block sampling/census for each wedge will be conducted in parallel with fieldwork. Sensitization will occur at the same time as mapping. Below, the mapping/sensitization team is referred to as mobilization/mapping team.

In advance of field mapping:

- Develop a list of randomly selected hamlets, draw on a geographic map, and develop a mapping plan to schedule the enumeration for the hamlets (list hamlets and organize based on proximity, and how many hamlets to be covered per day). See **“*Mama na Mtoto* Mapping Plan”;**
- Assign each hamlet a number and assign random numbers 1-8 for direction of transect line- corresponding to 8 directions: North=1, Northeast=2, East=3, Southeast=4, South=5, Southwest=6, West=7, Northwest=8;
- Develop mapping/sensitization forms and checklists ***See *Mama na Mtoto* Baseline Coverage Survey (2016) Households Enumeration Form;**
- Select and train mappers/sensitizers;
- Obtain a list of contacts for the VEO of each area to be mapped/sensitized;
- Determine the proximity of hamlets to one another for coordinating teams and transport for each mapping day;
- Identify one member of each mobilization/mapping team who will accompany the Ras, conducting data collection in the area they previously mapped 2-3 days ago, in order to act as a guide and facilitate the identification of households to be surveyed;
- Prepare supplies including GPS devices, stationary, and photos of tablets;
- Ensure that the VEO is contacted prior to the visit at least one day before mapping to give advanced notice of the mapping and to request an introduction to the Hamlet Leader and contact details for the Hamlet Leader (if available). This will help to avoid a long waiting time to meet with the appropriate person or not being able to contact the appropriate person that day. Confirm if any HH lists may be available for the hamlets. If it has rained/is raining, confirm whether the hamlet is still accessible by vehicle.

**Mapping/sensitization (mobilization) team visits each hamlet 2-3 days before RA teams:**

***See Sensitization Checklist for Mobilization/Mapping Research Team**

- Meet with the VEO and provide letters from the Regional Medical Officer and CUHAS (PI and Research Ethics office) about the study; provide information about survey details, timing, logistics, purpose, and mapping/sensitization to be conducted;
- Drive to the hamlet and note any landmarks to remember the route to the hamlet in preparation of leading the RAs for data collection 2-3 days later;
- Meet and identify hamlet leader and/or guide, confirm names and contact information;
- Note time of start of mapping on form and hamlet name;
- Identify team members and survey purpose, timing, logistics, details, and discuss the mapping/sensitization protocol (“*wedge approach*”);
- Confirm name of hamlet, village;
- Identify if in actual fact, hamlet is rural, urban or mixed (not just as listed). Make a note on the enumeration form if the hamlet is found to be classified incorrectly;
- Assess estimated HH numbers. Ask to see HH lists if available. Try to get best estimate of HH numbers for entire hamlet. Ensure definition of HH is clear. Should the estimated number of HHs be close to 30, consider enumerating the entire hamlet (this can be done up to 45 HHs);
- Inquire about specific dates of importance to hamlet i.e. markets, weddings, worship and any specific features of finding families and women at home (e.g. farming, mining, other) as well potential challenges (e.g. area not accessible by vehicle, households are located on either side of a mountain or swamps, etc.);
- Review general geography of hamlet-main features, boundaries, clusters of homes;
- Travel to geographic centre of hamlet (middle of the hamlet). *Note that the centre is often confused with areas of gathering, such as the market, etc. This may not be the same as the geographic middle of the hamlet. Record GPS coordinates. Take a photo with the GPS if possible either using the GPS (for those devices where this is available; this is best as the photo is taken along with GPS coordinates) or by phone. On the enumeration form, check the box off for “Photo taken at middle.” If the photo was taken with a phone, note whose phone was used for follow up;
- Turn on GPS mode for tracking travel route;
- Identify direction of planned travel (according to selected direction). Identify landmarks in intended direction;
- Walk/drive to periphery/boundary of hamlet in chosen direction. Note homes (dwellings) exactly on the line and to the right (clockwise);
- When boundary is reached, record GPS coordinates and turn off (or mark) end of transect line tracking on GPS unit. Ensure the tracking is saved. Take a photo with the GPS unit if possible. On the enumeration form, check the box off for “Photo taken at boundary.” If the photo was taken with a phone, note whose phone was used for follow up. Also check off “Transect line taken between the middle and the boundary”;
- Begin selection of 30 households closest and perpendicular to the line, using proportionally further distance the further out from the middle, ensuring coverage to the full boundaries as possible. Use a method that catches more distance in ‘blocks’ (see images’) in peripheries and little near the middle. If it is helpful in remembering the HHs, sketch a route with landmarks, to keep track of approximate location of the HHs, which will help to direct the RAs conducting data collection;
- At each household:
  1. Introduce team members and purpose (or hamlet leader/guide intro) of the visit, show identification. Be sure to communicate that that team is from ‘Bugando University’, as this would be familiar to people;
  2. Details of expected date and time of survey, logistics (how long it takes), who will be surveyed; explain data will be collected on a tablet (can show smart phone, and explain it is similar, like a computer);
  3. Identify within HH, the name of the HH head (and common name known by others), any women aged 15-49 years (including visitors, house girls, etc.), children U5, contact info for HH. Be sure to probe to determine the most accurate women aged 15-49 as well as children U5. It is advisable to be specific in asking about children’s ages (i.e. whether there are children who are ages 4, 3, 2, 1, or younger);
  4. Be sure to probe for potential multiple households in a dwelling/compound (who makes decisions and supports members of the family, where people sleep, whether people eat together, etc.). For large households, please make a note on the enumeration form so that data collection team can plan accordingly;
  5. Assign the dwelling number (1, 2, 3, etc.) and household. There should be 1- 30 households, up to 45 maximum. Households that share a dwelling should be numbered identified from 1-10 for each dwelling:

#1 D1, H1

#2 D1, H2

#3 D2, H1

#4 D3, H1

#5 D3, H2

etc…

- 1. Record the GPS coordinates, description of dwelling location;
  2. Make any additional notes for the dwelling that would help the data collection team to prepare for the interview e.g. whether the household does not have any Swahili speakers;
  3. If HH member not present, try to collect information from neighbors or the guide, and ask the guide to communicate information about the survey these household members.
- Mapping/sensitizing team makes a note of any clusters of dwellings on their map they skip over for a specific reason or when the hamlet boundaries are unclear—this will help when the GPS coordinates are mapped to be sure all appropriate dwellings are visited and not missed with this technique and also note key landforms;
- Note the sweep of the area clockwise from the line should form a bicycle ‘spoke’ shape;
- Ensure complete census of 30 households. In cases where there are fewer than 30 households in a hamlet (or there are some that are impossible to access), note these on the enumeration form. For hamlets with more than 30 households, up to 45, households can be enumerated to cover the whole hamlet;
- Mapping/sensitizing teams should make note of any anomalies they encounter during the mapping which make it difficult to complete according to these guidelines and should seek permission from their supervisor if they deviate. Any changes in protocol should be explained and documented and reviewed the same day with the study coordinator and reported to the PI/co-PI;
- Record finish time of mapping;
- Ensure all forms have correct hamlet label, HH numbers are in order and pages are numbered;
- Thank hamlet leader/guide, review plans for follow up and answer any outstanding questions/concerns, pay honorarium (5,000 TSH per guide; 15,000 TSH maximum per hamlet);
- Submit form(s) to Mobilization/Mapping Supervisor daily for review;
- Conduct a Mobilization/Mapping team debrief:

1.       What went well today?

2.       What were the challenges?

3.       What can we improve?

a. Provide any additional training if needed i.e. reviewing definitions, ensuring that common areas of challenge are addressed and concepts/protocol are understood

4.       Any Other Business

a.       Updates to the schedule (any changes, confirm time of departure, etc)

b.       Issues related to welfare, payment, etc. (e.g. accommodation, contracts)

5.       Appreciation of the team

- Participate in the debrief with the data collection team to obtain feedback on mobilization and mapping, following the format as above. The Mobilization/Mapping Supervisor should communicate any important information from the day’s mobilization/mapping (e.g. if the day scheduled for data collection is a day where the community is not available- weddings, burials, etc.);
- The Mobilization/Mapping Supervisor is to submit the forms to the *Mama na Mtoto* Logistics Supervisor, who is to photocopy each completed enumeration form and provide the copy to the Data Collection Coordination Team;
- The Logistics Supervisor is then to scan the forms and send to the Research Coordinators, along with the GPS tracking line, and the photos for the hamlet middle and boundary. Ensure that photos are named with an “M” to indicate the middle of the hamlet, and “B” to indicate the boundary of the hamlet;
- Ensure that the Mapping Plan is updated with the date mapping/sensitization was completed as well any important comments to note about hamlet, including any deviations to the protocol, where indicated;

Following submission of the maps and GPS coordinates, we will aim to map the coordinates and hope to see the shaping like the expected wedges and hope the transect lines to approximately directional (we appreciate landforms may not allow travel this way, that should be shown easily on google maps).

During this exercise, on some selected days, we will plan for a study coordinator to do unannounced ‘checks’ on one or more maps for each mapping team. This check should be documented. If there are concerns raised by the shapes or apparent missed dwellings on google maps, these checks should be more frequent.

Original copies of the enumeration forms will be kept in a secure file and stored by the Logistics Supervisor. The Logistics Supervisor will also keep a file of the scanned enumeration forms, an updated Mapping Plan, tracking information from the GPS, and photos for the middle and boundary.

**RA team visit to the hamlet:**

- A member of each mapping/sensitizing team travels with RAs to the hamlet and shows them the boundaries of the block and helps to guide them to households which were registered during mapping
- RAs are assigned to specific households
- New (non-listed) HHs are numbered starting at 101 for RA 1, 201 for RA 2, and so on. (will write script to renumber in the database so that we have HHs consecutively numbered from 1 to n)

http://ije.oxfordjournals.org/content/33/3/469.fullhttp://gametlibrary.worldbank.org/FILES/1338_LQAS%20vs%20Cluster%20Sampling%20-%20descriptions%20of%20both.pdf
